# Microbiota control acute arterial inflammation and neointimal hyperplasia development after arterial injury

**DOI:** 10.1371/journal.pone.0208426

**Published:** 2018-12-06

**Authors:** Kelly Wun, Betty R. Theriault, Joseph F. Pierre, Edmund B. Chen, Vanessa A. Leone, Katharine G. Harris, Liqun Xiong, Qun Jiang, Melanie Spedale, Owen M. Eskandari, Eugene B. Chang, Karen J. Ho

**Affiliations:** 1 Division of Vascular Surgery, Northwestern University, Chicago, IL, United States of America; 2 Department of Surgery and Animal Resources Center, University of Chicago, Chicago, IL, United States of America; 3 Department of Pediatrics, The University of Tennessee Health Science Center, Memphis, TN, United States of America; 4 Department of Medicine, Section of Gastroenterology, University of Chicago, Chicago, IL, United States of America; Nagoya University, JAPAN

## Abstract

**Background:**

The microbiome has a functional role in a number of inflammatory processes and disease states. While neointimal hyperplasia development has been linked to inflammation, a direct role of the microbiota in neointimal hyperplasia has not yet been established. Germ-free (GF) mice are an invaluable model for studying causative links between commensal organisms and the host. We hypothesized that GF mice would exhibit altered neointimal hyperplasia following carotid ligation compared to conventionally raised (CONV-R) mice.

**Methods:**

Twenty-week-old male C57BL/6 GF mice underwent left carotid ligation under sterile conditions. Maintenance of sterility was assessed by cultivation and 16S rRNA qPCR of stool. Neointimal hyperplasia was assessed by morphometric and histologic analysis of arterial sections after 28 days. Local arterial cell proliferation and inflammation was assessed by immunofluorescence for Ki67 and inflammatory cell markers at five days. Systemic inflammation was assessed by multiplex immunoassays of serum. CONV-R mice treated in the same manner served as the control cohort. GF and CONV-R mice were compared using standard statistical methods.

**Results:**

All GF mice remained sterile during the entire study period. Twenty-eight days after carotid ligation, CONV-R mice had significantly more neointimal hyperplasia development compared to GF mice, as assessed by intima area, media area, intima+media area, and intima area/(intima+media) area. The collagen content of the neointimal lesions appeared qualitatively similar on Masson’s trichrome staining. There was significantly reduced Ki67 immunoreactivity in the media and adventitia of GF carotid arteries 5 days after ligation. GF mice also had increased arterial infiltration of anti-inflammatory M2 macrophages compared to CONV-R mouse arteries and a reduced proportion of mature neutrophils. GF mice had significantly reduced serum IFN-γ-inducible protein (IP)-10 and MIP-2 5 days after carotid ligation, suggesting a reduced systemic inflammatory response.

**Conclusions:**

GF mice have attenuated neointimal hyperplasia development compared to CONV-R mice, which is likely related to altered kinetics of wound healing and acute inflammation. Recognizing the role of commensals in the regulation of arterial remodeling will provide a deeper understanding of the pathophysiology of restenosis and support strategies to treat or reduce restenosis risk by manipulating microbiota.

## Introduction

Peripheral arterial disease (PAD) is a burgeoning global health problem due to lifestyle-related risk factors, aging of the population, and increasing prevalence of risk factors such as diabetes mellitus and hypertension.[1] Symptomatic PAD can be treated with endovascular and surgical modalities, but restenosis secondary to neointimal hyperplasia, which occurs in the first 6–18 months, is a pervasive problem that leads to reinterventions, worse patient survival, and risk of limb loss. This is a process that is distinct from progression of atherosclerosis, which occurs over years.[2] Despite advances in primary and secondary treatment for PAD, the prevention and durable treatment of neointimal hyperplasia remain elusive.[3, 4]

Neointimal hyperplasia is the result of arterial “injury” manifested by creation of a surgical anastomosis, balloon dilation, or stent implantation. These modes of endothelial injury induce a wound healing response that is driven by multiple biochemical and cellular factors[4], including local platelet adherence and aggregation, fibrinogen binding, thrombus formation, and activation of an inflammatory cascade that modulates smooth muscle cell migration, extracellular matrix production, and cellular proliferation.

The gut microbiome has a functional role in a number of inflammatory processes and disease states,[5] in the development of the immune system,[6, 7] and in wound healing.[8] While neointimal hyperplasia development has been linked to local and systemic inflammation,[9–12] and while our prior work demonstrated that modulation of the gut microbiome with antibiotics modulated neointimal hyperplasia in a rat model of carotid angioplasty,[13] direct demonstration of the role of the gut microbiome in neointimal hyperplasia has not yet been made.

Germ-free (GF), or axenic, mice are born and raised in sterile isolators and are completely lacking in all microbiota.[14] They are an invaluable model for studying causative links between commensal organisms and the host, since observed phenotypes resulting from resident microbiota or from host-microbiota interactions can only be distinguished by comparing the same phenotypes in animals lacking all microbiota, with all other factors being equal. Thus, we hypothesized that GF mice, which have attenuated inflammation with age,[15] in colitis,[16, 17] wound healing,[8] and diabetes mellitus[18, 19] would have reduced local and systemic inflammation and neointimal hyperplasia development following carotid ligation compared to conventionally raised (CONV-R) mice.

## Methods

### Experimental animals

Twenty-week-old GF mice were bred and maintained in The University of Chicago Gnotobiotic Research Animal Facility. Mice were housed in positively pressured flexible film isolators under a 12-hour light cycle and fed autoclaved chow and water *ad libitum*. All procedures on GF mice were approved and conducted in accordance with The University of Chicago Institutional Animal Care and Use Committee.

The comparison cohort consisted of conventionally raised (CONV-R) 20-week-old male C57BL/6 mice (Jackson Laboratories, Bar Harbor, ME) housed in a conventional facility at Northwestern University under a 12-hour light cycle. Standard irradiated mouse chow and autoclaved drinking water were provided *ad libitum*. Experiments involving these mice were approved and conducted in accordance with the Northwestern University Animal Care and Use Committee.

All animals in this study were treated in accordance with the Guide for the Care and Use of Laboratory Animals published by the National Institutes of Health.

### Unilateral carotid artery ligation and tissue collection

All animals underwent left carotid artery ligation as previously described [20]. In brief, mice were anesthetized with ketamine and xylazine. A longitudinal paramedian incision was made over the left carotid artery. Blunt dissection was used to separate the jugular vein and vagus nerve from the left common carotid artery. An 8–0 Prolene ligature was placed around the distal common carotid artery just proximal to the carotid bifurcation.

At the appropriate time point, mice were euthanized and *in situ* perfusion fixation with phosphate-buffered saline (PBS) and 2% paraformaldehyde via cardiac puncture was performed prior to harvest of both carotid arteries. The right common carotid artery served as the unoperated control. Arteries were fixed in 2% paraformaldehyde at 4°C for one hour followed by overnight cryoprotection in 30% sucrose at 4°C and then embedded in OCT compound (Tissue-Tek, Sakura Finetek, Torrance, CA). Whole blood was collected by cardiac puncture prior to perfusion fixation at the time of sacrifice. Serum was isolated and stored at -80°C until use.

### Surgical procedures in GF mice and monitoring for sterility

GF mice were transported out of the sterile flexible film isolator in a manner previously described to an EdgeGard laminar flow hood (The Baker Company, Stanford, ME), which was used for the surgical procedure.[21] The laminar flow hood has 100% high-efficiency particulate arrestance filtered air delivered to the working area. Interior surfaces were sprayed with chlorine dioxide solution and then custom-made drapes sterilized by autoclaving or ethylene oxide were used to cover the dissecting microscope and the inner working area of the BSC. The surgical procedure was performed as described above for CONV-R mice with the surgeon wearing a sterile gown and gloves. At the conclusion of the surgery, GF mice were transported back to the sterile flexible film isolator in a sterile fashion. Stool pellets were collected weekly and used for cultivation in aerobic and anaerobic conditions and for DNA extraction and 16S rRNA qRT-PCR as previously described.[21]

### Tissue processing and morphometric analysis

Serial five-μm frozen sections were collected on Superfrost Plus slides (Fisher Scientific, Pittsburgh, PA) from just distal to the carotid bifurcation to the aortic arch, air dried, and stored at -20°C. Carotid arteries were examined for neointimal hyperplasia after hematoxylin-eosin staining of artery sections at evenly-spaced 350-μm intervals between the carotid bifurcation and the aortic arch. Masson’s trichrome staining was also performed to visualize arterial wall structures. Digital images of stained sections were obtained using a Leica DM2000 light microscope with a 20x objective and camera (W. Nuhsbaum, Inc., McHenry, IL). Morphometric analysis was performed by using lumen, intima, and media areas and circumference that were measured or calculated using ImageJ/FIJI software (NIH, Bethesda, MD) after calibration.

### Immunofluorescence and quantification

Rabbit polyclonal antibody to Ki67, rabbit monoclonal antibody to α-smooth muscle actin (SMA) (E184), rat monoclonal antibody to CD45 (IBL-3/16), rabbit polyclonal antibody to CD206, rat monoclonal antibody to NIMP (NIMP-R14), and rabbit polyclonal anti-liver arginase (Arg1). Mouse monoclonal antibody to CD68 (MCA341R) was purchased from Bio-Rad (Hercules, CA). Rat monoclonal antibody to Ly6G (1A8) was purchased from BD Biosciences (San Jose, CA). Secondary antibodies were Alexa Fluor 555 goat anti-rabbit IgG, goat anti-mouse IgG, goat anti-rat IgG from Invitrogen (Waltham, MA) and Cy3 donkey anti-rat from Jackson ImmunoResearch Laboratories (West Grove, PA). Frozen sections were outlined using ImmEdge hydrophobic barrier pen from Vector Labs (Burlingame, CA), rehydrated, fixed in 2% paraformaldehyde, washed with PBS, incubated with the primary antibodies diluted in IHC-Tek diluent pH 7.4 from IHC-World (Woodstock, MD) for 1 hour at room temperature, washed in PBS, incubated with the appropriate secondary antibody diluted in IHC-Tek diluent pH 7.4 for 1 hour at room temperature, washed in PBS and water, and mounted with ProLong Gold Antifade mountant with DAPI from ThermoFisher Scientific (Waltham, MA). For Ly6G staining, slides were fixed in acetone at 20°C for 10 minutes, air dried for 15 minutes, washed in PBS, incubated with primary antibody overnight at 4°C, washed in PBS, and incubated with the appropriate secondary antibody.

Primary antibodies were used at the following concentrations: Ki67, 2 μg/mL; CD45, 1 μg/mL; CD68, 2 μg/mL; CD206, 2 μg/mL; NIMP, 1 μg/mL; Arg1, 5 μg/mL; Ly6G, 0.5 μg/mL; and α-SMA, 0.2 μg/mL. AlexaFluor 555 goat anti-rabbit IgG secondary antibody concentration was 4 μg/mL for secondary staining of Ki67- and Arg1-immunostained sections, and was 2 μg/mL for CD206- and α-SMA-immunostained sections. AlexaFluor 555 goat anti-rat secondary antibody concentration was 2 μg/mL for CD45- and NIMP-R14-immunostained sections, and AlexaFluor 555 goat anti-mouse IgG secondary antibody concentration was used at 2 μg/mL for CD68-immunostained sections. Cy3 donkey anti-rat secondary antibody concentration was 1.5 μg/mL for L6G staining.

Digital images were acquired using a Nikon Eclipse 50i microscope (Nikon Instruments, Inc., Melville, NY) with a 20x objective and equipped with DAPI, Texas red, and FITC emission filters, SPOT Advanced Imaging Software (Diagnostic Instruments, Sterling Heights, MI), and a RT KE Color 3-Shot digital microscope camera (Diagnostic Instruments, Sterling Heights, MI).

Positively-stained cells and DAPI-stained nuclei in the intima, media, and adventitial layers of each high-powered field were manually counted in a blinded fashion using FIJI/ImageJ Cell Counter plugin (NIH, Bethesda, MD), and a ratio of immunoreactive cells per total cells in each high-powered field was calculated as the staining index. Ten non-overlapping high-powered fields (.26 mm^2^) per arterial wall layer (intima, media, and adventitia) in each of six arterial sections sampled in evenly spaced intervals along the entire length of the carotid arteries from eight to ten animals per treatment group were examined and used for quantification.

Neointimal α-SMA signal intensity was quantified using FIJI/ImageJ, normalized to the neointimal area, and expressed as a percentage. The minimum signal thresholds were determined from negative controls; the maximum thresholds were the upper allowable limit of the image bit-depth. A total of 14 to 17 arterial sections were assessed in each group.

### Multiplex immunoassays

The Cytokine and Chemokine Convenience 26-Plex Mouse ProcartaPlex Panel from Invitrogen (Waltham, MA) was used to measure the serum concentration of 26 cytokines, chemokines, and growth factors [IL-1β, IL-2, IL-4, IL-5, IL-6, IL-9 IL-10, IL-12p70, IL-13, IL-17A, IL-18, IL-22, IL-23, IL-27, IFN-ɣ-inducible protein (IP)-10, MCP-1, MCP-3, MIP-1α, MIP-1β, MIP-2, TNF-α, IFN-γ, GRO-α, GM-CSF, RANTES, and eotaxin]. The assay was performed using the MAGPIX microplate reader by Luminex (Austin, TX) according to the manufacturer’s instructions.

### Statistical analysis

Summary data were expressed for each group as mean ± SEM. Statistical significance in pairwise comparisons was tested with an unpaired Student’s t-test, and results with P < .05 were considered significant. All data were analyzed using Prism 7 (GraphPad, La Jolla, CA).

## Results

### GF mice have attenuated neointimal hyperplasia development after carotid ligation compared to CONV-R mice

**Fig 1:**
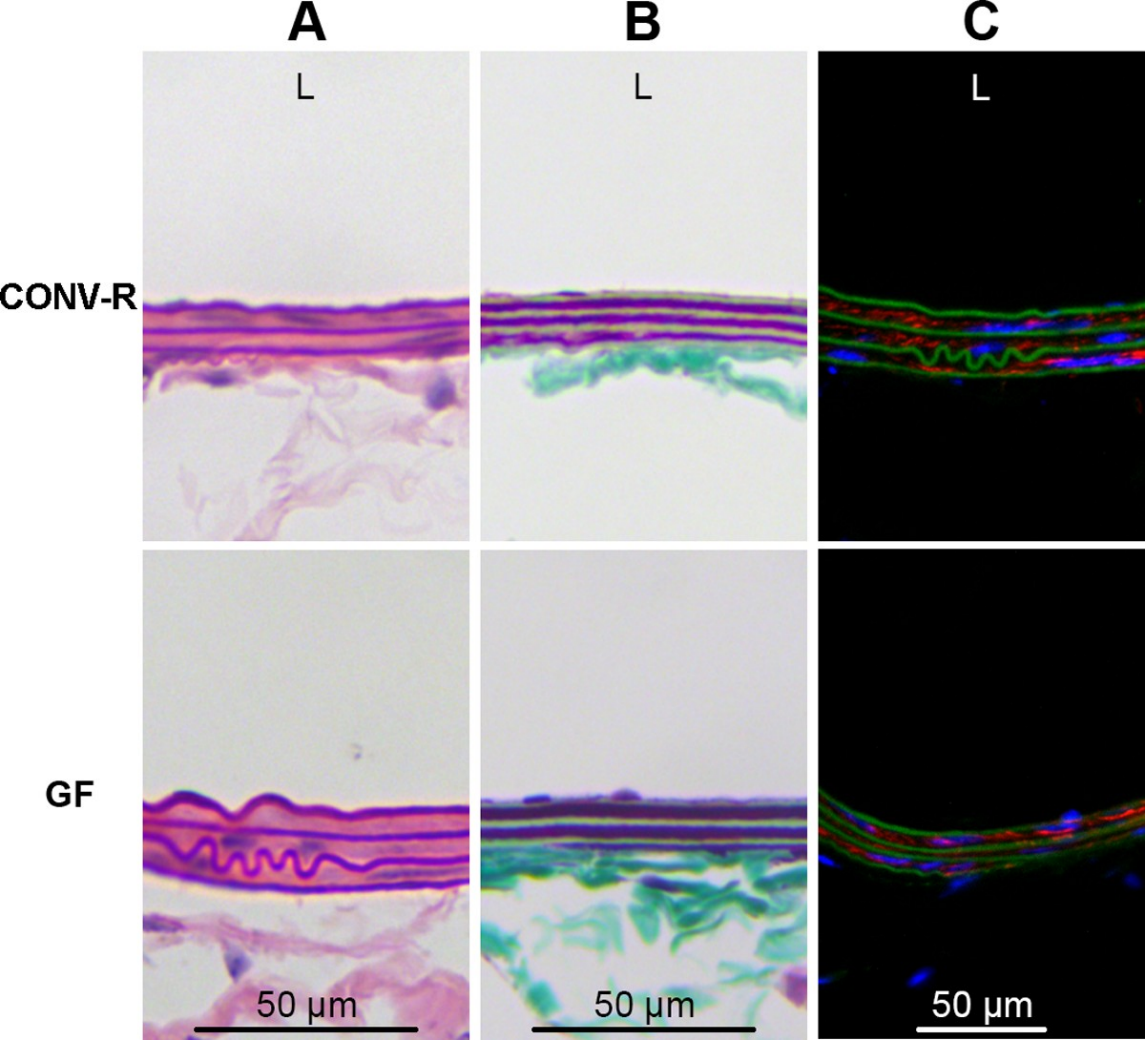
CONV-R and GF carotid arteries are similar at baseline. Conventionally-raised (CONV-R) and germ-free (GF) carotid arteries appeared qualitatively similar after (*A*) hematoxylin and eosin staining and (*B*) Masson’s trichrome staining. (*C*) α-SMA immunofluorescence was also qualitatively similar between CONV-R and GF arteries. Blue represents DAPI staining; red represents α-SMA; green is autofluorescence of the elastic lamina. Six mice per group were examined. Original magnification 200x. L, lumen side of artery.

The carotid arteries of CONV-R and GF mice without surgical intervention were grossly similar in external appearance. There were no qualitative differences in the histologic appearance of the uninjured right carotid arteries after H&E staining and Masson’s trichrome staining (Fig 1A and 1B). Furthermore, there were no qualitative differences in α-SMA immunofluorescence in the uninjured carotid arteries of CONV-R and GF mice (Fig 1C).

**Fig 2:**
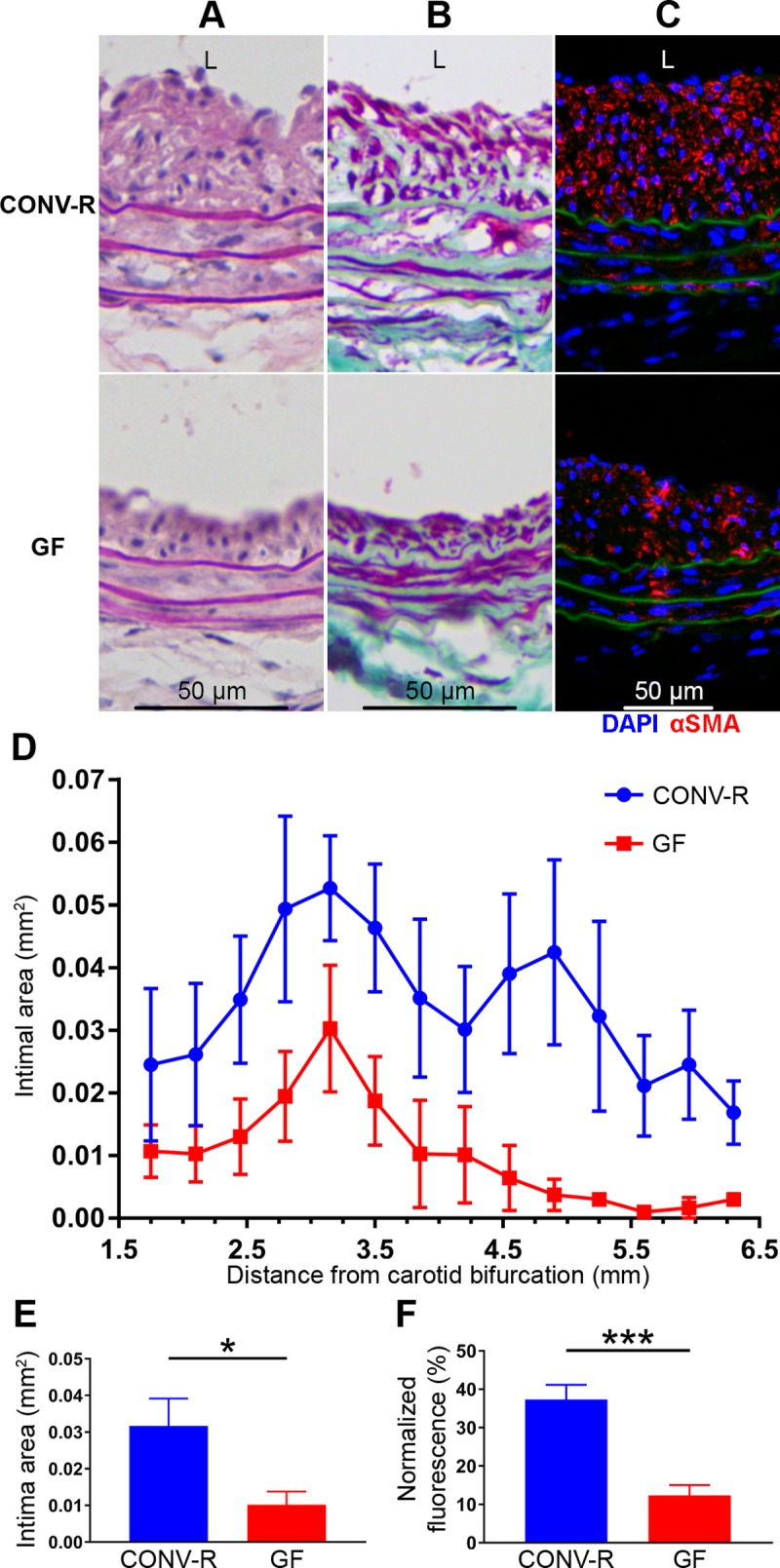
Neointimal hyperplasia development in carotid arteries 28 days after ligation is attenuated in GF mice compared to CONV-R mice. Representative H&E staining of the post-injury carotid arteries is shown in (*A*), demonstrating greater neointimal hyperplasia development in CONV-R mice compared to GF mice. Representative Masson’s trichrome staining of post-injury arteries is shown in (*B*), demonstrating qualitatively similar collagen content of the neointima. α-SMA immunofluorescence (red) was significantly less in the GF carotids; blue represents DAPI staining; green represents autofluorescence of elastin (*C*). Mean intima area at intervals of 350 μM across the length of the common carotid artery from the carotid bifurcation to the aortic arch demonstrates significantly more neointimal hyperplasia in CONV-R mice than in GF mice at every interval (*D*), P = .01. (*E*) Mean overall intima area across the length of the common carotid artery shown in *(D)* was significantly greater in CONV-R than in GF mice, .032±.007 mm^2^ vs. .010±.004 mm^2^, P = .04 (*). (*F*). α-SMA fluorescence was normalized to intima area and was observed to be significantly greater in CONV-R than in GF mice, 37.3±3.8% vs. 12.3±2.7%, P < .001 (***). Original magnification of all photomicrographs 200x. N = 5–10 mice per group. L, lumen side of artery.

Twenty-eight days after left carotid ligation, CONV-R mice had significantly more neointimal hyperplasia development compared to GF mice, as assessed by intima area, media area, intima+media area, and intima area/(intima+media area) (Fig 2A, 2D and 2E and S1 Table). The collagen content of the neointimal lesions appeared qualitatively similar on Masson’s trichrome staining (Fig 2B). However, α-SMA immunofluorescence in the neointima was significantly attenuated in GF carotid arteries compared to CONV-R carotid arteries (Fig 2C and 2F).

### Distribution of early arterial cellular proliferation after carotid ligation is altered in GF mice

**Fig 3:**
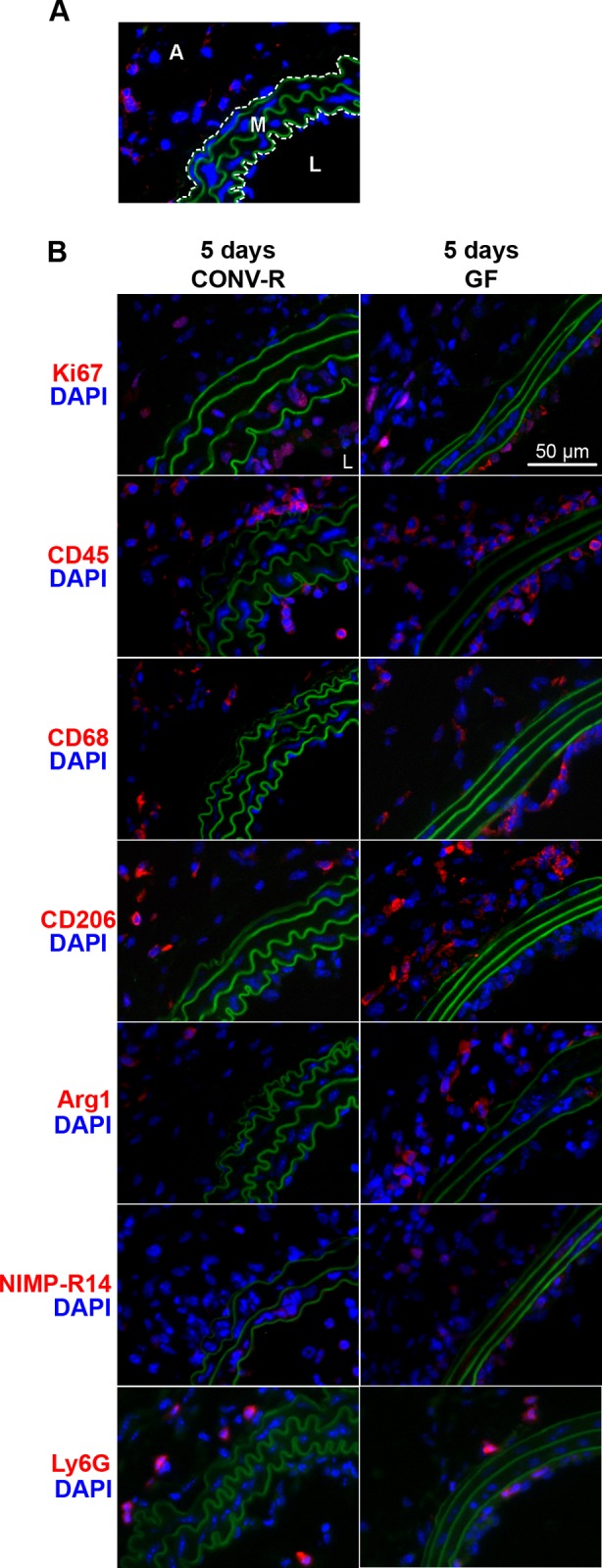
Ki67, CD45, CD68, CD206, Arg1, and NIMP-R14 immunofluorescence of carotid arteries after ligation in CONV-R and GF mice. (*A*) Sample high-powered field of an arterial section showing the orientation and layers of the arterial wall. The internal and external elastic laminae (which have green autofluorescence) have been outlined by dotted lines. The adventitial (A) and medial (M) layers outside and between the laminae, respectively, are shown. The lumen is denoted by L. (B) Representative immunofluorescence of Ki67, CD45, CD68, CD206, Arg1, NIMP-R14, and Ly6G 5 days after ligation in CONV-R and GF mice. All arterial sections are oriented in the same way as in (A) with the lumen in the bottom right-hand corner. Original objective magnification 200x for all images. N = 6–8 mice per group per time point.

**Fig 4:**
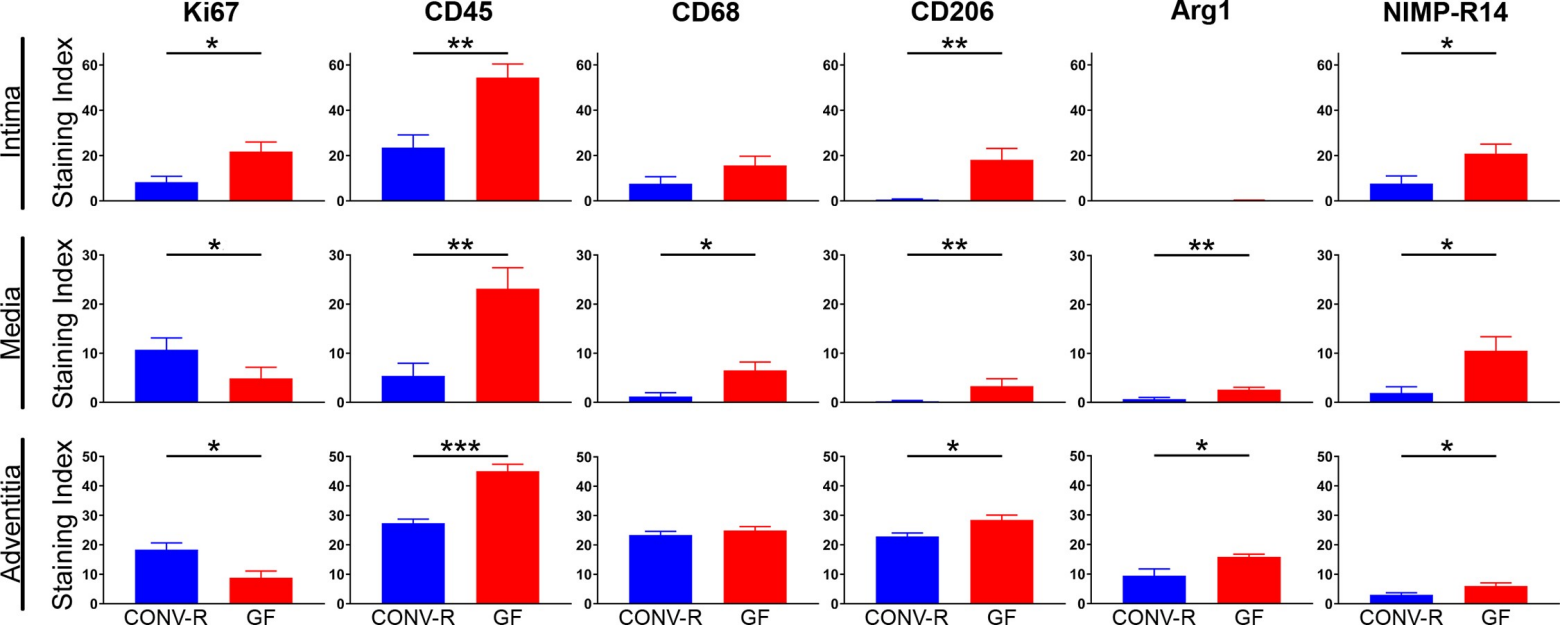
Quantitative comparison of Ki67, CD45, CD68, CD206, Arg1, NIMP-R14, and Ly6G immunofluorescence in each layer of carotid arteries 5 days after ligation in CONV-R and GF mice. Staining index for each cell marker was calculated as ratio of positively stained cells to total number of cells per high powered field (HPF). N = 110–150 unique HPFs per tissue layer per group. *, P≤.05. **, P≤.01. ***, P≤.001.

Cell proliferation was greatest in the medial and adventitial layers of the arterial wall 3–7 days out of days 1–7 after carotid ligation in CONV-R mice, as demonstrated by Ki67 immunoreactivity (data not shown). Thus, we chose the five-day time point to compare cell proliferation after carotid ligation in CONV-R and GF mice. At this time point, GF carotid arteries had similar overall Ki67 immunoreactivity compared to CONV-R arteries (6.6±1.1%, CONV-R vs. 9.7±2.0% GF, P = .24), but the distribution of Ki67-positive cells in each layer of the arterial wall differed between the two groups. There was greater Ki67 staining in the intima of GF mice (P = .03) but decreased staining in both the medial and adventitial arterial layers compared to CONV-R mice (P = .03 and P = .01, respectively) (Figs 3A and 4) (S2 Table).

### Differential local arterial inflammatory response in GF and CONV-R mice

Neointimal hyperplasia development after carotid ligation is accompanied by acute local inflammation within the arterial wall.[20, 22] As CD45 is a cell surface protein found on all leukocytes,[23] we next examined CD45 immunofluorescence in the arterial wall, followed by CD68 (macrophage marker), and Arg1 and CD206 (M2 macrophage markers) at 1, 3, 5, and 7 days after carotid ligation in CONV-R mice.

When we quantified immunofluorescence of CD45, CD68, and CD206 at all four early time points in both groups of mice by calculating staining indices (schematic of arterial sections shown in Fig 3A), we found that staining of all three markers peaked by 5 days (data not shown). Thus, we proceeded to compare immunofluorescence of CD45, CD68, CD206, Arg1, NIMP-R14 (neutrophil marker[24]), and Ly6G (mature neutrophil marker[25]) in post-ligation arteries of CONV-R and GF mice at 5 days. As shown in Fig 4 and S2 Table, the staining indices of CD45-, CD206-, and NIMP-R14 in the intimal layer was significantly higher in GF arteries than CONV-R arteries (P = .003, P = .005, and P = .5, respectively), while there was no significant difference in the staining index of CD68 between the mouse groups. In the medial layer (Fig 4 and S2 Table), staining indices of CD45, CD68, CD206, Arg1, and NIMP-R14 were all higher in GF mice. The absolute numbers of CD45-, CD68-, CD206-, Arg1-, and NIMP-R14-positive cells were highest in the adventitial layer of both mouse groups at the five-day time point compared to the intimal and medial layers (data not shown). Lastly, as seen in the adventitial layer, the staining indices of CD45, CD206, Arg1, and NIMP-R14 were higher in GF mice compared to CONV-R mice (P < .001, P = .02, P = .05, and P = .04, respectively), while there was no difference in the adventitial staining index of CD68 (P = .46). There were no significant differences in the Ly6G staining indices between CONV-R and GF mice in any arterial layer (Fig 4 and S2 Table), suggesting that the proportion of mature/differentiated neutrophils is diminished in GF mice compared to CONV-R mice.

### Distinct systemic chemokine profiles after carotid ligation in GF and CONV-R mice

**Fig 5:**
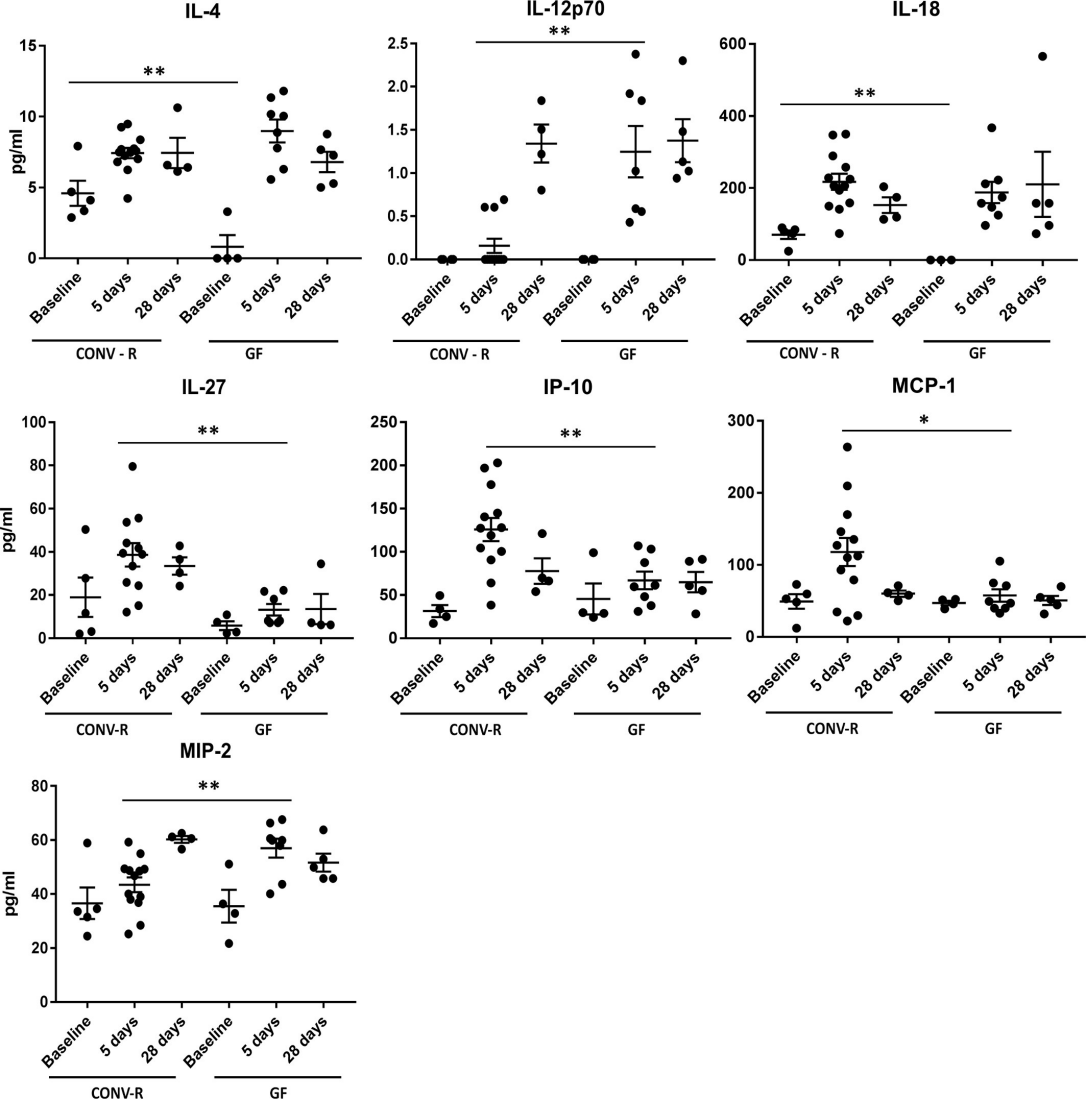
Serum concentrations of selected cytokines in CONV-R and GF mice at baseline and at five and 28 days after carotid ligation. CONV-R mice had significantly elevated IL-4 and IL-18 at baseline than GF mice (P = .03 and P = .04, respectively). Five days after carotid ligation, CONV-R mice had greater increases in IL-27 and IP-10 over baseline than GF mice (P = .001 and P = .004, respectively) but blunted augmentation of IL-12p70 and MIP-2 (P = .005 and P = .006, respectively). By 28 days, serum levels of all assessed cytokines and chemokines were similar to baseline levels in both CONV-R and GF mice. *, P = .05; ** P < .05.

GF mice had significantly different serum chemokines and cytokine profiles compared to CONV-R mice. At baseline, CONV-R mice had significantly elevated serum levels of IL-4 and IL-18 than their GF counterparts (IL-4: 4.59±.89 pg/ml CONV-R vs. .82±.82 pg/ml, P = .03; IL-18: 70.36±11.71 pg/ml CONV-R vs. undetectable in GF, P = .04) (Fig 5). Five days after carotid ligation, CONV-R had significantly greater increases in serum concentration of IL-27 and IP-10 than GF mice (IL-27: 38.66±5.42 pg/ml CONV-R vs. 13.24±2.67 pg/ml GF, P = .001; IP-10: 125.8±13.42 pg/ml CONV-R vs. 67.02±10.27 pg/ml GF, P = .004). The greater increase in serum concentration of MCP-1 in CONV-R mice compared to GF mice at this time point did not reach statistical significance (117.9±19.55 pg/ml CONV-R vs. 57.53±8.61 pg/ml GF, P = .05). Conversely, at 5 days, CONV-R mice had lower augmentation in IL-12p70 and MIP-2 over baseline compared to GF mice (IP-12p70: .38±.23 pg/ml CONV-R vs. 1.75±.57 pg/ml GF, P = .005; MIP-2: 43.41±2.74 pg/ml CONV-R vs. 56.99±3.52 pg/ml GF, P = .006). By 28 days, there were no significant changes in cytokine and chemokine concentrations compared to baseline in either CONV-R or GF mice. Cytokine and chemokine concentrations for all time points can be found in S3 Table.

## Discussion

Although there is mounting literature investigating the impact of gut microbial community composition, diversity, and functional metabolomics output on health and disease,[26] on modulation of the immune system,[5, 7] atherosclerosis,[27, 28] hypertension,[29] platelet reactivity,[30, 31] and endothelial function,[32] the influence of microbiota on the arterial injury response in peripheral arteries after surgery is largely unexplored. The present report demonstrates the impact of microbiota on systemic and local arterial inflammation and on neointimal hyperplasia development after carotid ligation by a comparative study in GF and CONV-R mice. Other investigators have examined the causative role of microbiota in hypertension[29, 33] and stroke,[34, 35] thus providing evidence for the impact of microbiota on vascular beds which, unlike the skin or intestine, are not generally in direct contact with microbiota, To the best of our knowledge, this work provides the first direct evidence for the role of microbiota in the innate propensity to develop restenosis in contrast to what occurs in the presence of microbiota or of host-microbiota interactions.

Neointimal hyperplasia is a complex orchestration of cellular and biochemical events: there is an immediate response to endothelial denudation, exposure of subintimal matrix components, and vessel dilation. This is followed by platelet adherence and aggregation, thrombus formation, migration and proliferation of smooth muscle cells, inflammatory cytokine and chemokine signaling, extracellular matrix synthesis, and finally, fibroplasia and scar formation.[4] The mouse carotid ligation model of neointimal hyperplasia is well-described, readily reproducible, and is characterized by medial smooth muscle cell de-differentiation, proliferation, and migration that forms a substantive neointima with constrictive remodeling in the ipsilateral artery within 28 days. While the stimulus for remodeling is flow cessation with an intact endothelium[36] which does not directly mimic what occurs in human arteries after procedures such as angioplasty, stenting, or bypass grafting, the VSMC proliferative and migratory response and local arterial inflammatory reaction[9, 20, 22, 37, 38] are similar, and hence this model been widely used for elucidation of the factors controlling VSMC behavior in response to injury.[36]

We found that GF mice have attenuated neointimal hyperplasia development compared to CONV-R mice, which correlated with less local vascular smooth muscle cell (VSMC) proliferation in the media and adventitia. Since neointimal hyperplasia can be regarded as an exuberant wound healing response,[39] our finding that GF mice had less neointimal hyperplasia development is consistent with prior reports of accelerated wound closure and decreased scar formation in skin wounds,[8] decreased intestinal and lung pathology in a model of intestinal ischemia/reperfusion,[40] and attenuated colitis[16, 17] in GF mice.

Like others, we observed that although the carotid artery ligation model features an intact endothelium, a local arterial inflammatory response characterized by proinflammatory cytokine and neutrophil and macrophage accumulation can be detected during the first few days.[9, 20, 22, 37, 38] We observed a peak in local arterial leukocyte accumulation in CONV-R mice at 5 days, which corroborates prior investigations demonstrating a steady rise in leukocyte infiltration up to 7 days.[38] Early leukocyte accumulation in both CONV-R and GF mice was greatest in the adventitial layer, consistent with prior reports by others demonstrating that early adventitial accumulation of macrophages were required for constrictive remodeling,[41] that macrophage depletion prevents flow-mediated remodeling after partial carotid ligation,[42] and that inward remodeling was associated with transient adventitial macrophage activation and superoxide-stimulated cytokine production.[42] However, in the current study, GF mice had higher proportions of cells with an M2 macrophage marker than CONV-R mice, suggesting that microbiota influence both the accumulation and polarization of macrophages at the local site of arterial injury. M2 macrophages are referred to as anti-inflammatory “repair” macrophages, in contrast to classical pro-inflammatory M1 macrophages and are associated with wound healing and angiogenesis.[43, 44] Our observation corroborates the observation that GF mice with skin wounds had earlier and greater macrophage wound infiltration, greater levels of macrophage activity in the wound, and predominance of cells with the M2 phenotype, [8] suggesting a more favorable repair process in the absence of microbes.

Consistent with the above findings, we observed that GF mice also had decreased circulating MCP-1 and IP-10 at 5 days compared to CONV-R counterparts. MCP-1 is a pro-inflammatory cytokine and chemoattractant for monocytes and macrophages. Previous investigators have shown that in a mouse model of cuff-induced arterial injury, inhibition of MCP-1 activity led to decreased monocyte infiltration, attenuated early vascular inflammation, and diminished neointimal hyperplasia development.[45] Furthermore, in a rat model of carotid artery injury, inhibition of MCP-1 activity led to a reduction in VSMC number in the neointima and decreased overall neointimal formation.[46] Similarly, IP-10 is a chemokine secreted from multiple cell types after inflammatory (*e*.*g*. IFN-g or LPS) stimulation[47, 48] and is a monocyte and T cell chemoattractant.[49] RNAi inhibition of IP-10 expression blocked VSMC migration and attenuated the development of neointimal hyperplasia in a rabbit model of carotid artery injury.[50] Furthermore, IP-10 has been shown to be a potent mitogenic and chemotactic factor for VSMC with increased gene expression in the rat carotid artery after balloon angioplasty,[51] suggesting an active role for IP-10 in vascular remodeling. These findings are in concordance with our observation of decreased neointimal hyperplasia development in GF mice, which have less augmentation of MCP-1 and IP-10 after arterial injury compared to CONV-R mice.

In general, the early inflammatory response following arterial injury is characterized by early influx of neutrophils,[52] and neutrophil transmigration has been reported in the development of carotid atherosclerosis[53] and in the response to endothelial denudation induced by balloon angioplasty.[54] In hyperlipidemic mice, partial carotid ligation (a model similar to ours) results in early granulocyte arterial accumulation that peaks in a bimodal distribution at 4 days and at 14 days.[38] We hypothesized that GF mice would have less neutrophil accumulation than CONV-R mice, since GF mice with skin wounds have delayed and lower magnitude neutrophil infiltration compared to CONV-R mice,[8] and GF mice are known to be neutropenic compared to CONV-R mice[8, 55], suggesting that neutropenia and/or neutrophil dysfunction in GF mice accounts for reduced infiltration of neutrophils into healing wounds. In contrast, we observed increased total neutrophil accumulation in GF mice. However, GF mice had similar abundance of mature (differentiated) neutrophils between GF and CONV-R. In combination with increased serum MIP-2 concentration (a neutrophilic chemokine) in GF mice at the 5-day time point compared to CONV-R mice, we infer that there is a greater proportion of inactive or dysfunctional neutrophils in the GF mice secondary to impaired neutrophil stimulatory pathways.

Like other investigators, we observed that GF mice have lower baseline levels of IL-4 compared to CONV-R mice.[56] Similarly, since the IL-23, IL-22, and IL-18 axis is involved in host defense and in mediating and regulating inflammatory immune responses upon parasitic and bacterial infection,[57–60] it is conceivable that GF mice would have lower circulating levels of IL-18 at baseline. Interestingly, while some investigators observed decreased intestinal IL-10 expression[61–63] and decreased tissue expression of IL-10 in skin wounds in GF mice,[8] others have shown that GF mice have an attenuated local and systemic response to intestinal ischemia/reperfusion injury that corresponds to marked increases in endogenous IL-10 production at both the local (intestines) and remote (lung) tissue level[40]. In contrast with these other studies, our disease model involves peripheral arteries which are not typically in direct contact with microbiota. We observed increases in IL-10 at both 5 and 28 days after arterial injury compared to baseline in both GF and CONV-R mice, but the degree of augmentation did not differ significantly between them.

Gut microbiota are known to play a pivotal role in the development of the immune system. GF mice are known to have defects in splenic and mesenteric lymph nodes and show reduced Treg cell induction, reduction of Th17 cells, and Th1/Th2 imbalance, which is biased toward the Th2 response.[64, 65] This is consistent with our finding that at 5 days, GF mice have less augmentation of IL-27, which is involved in Th1 induction from naïve T cells.[66] Similarly, we and other investigators observed increased IL-12 (IL-12p70) in GF mice compared to CONV-R mice.[67] Since IL-12 is required for IFN-γ production, which is critical for the induction of Th1 cells,[56] these data add credence to the hypothesis that the GF mice have an immune response biased towards the Th2 response.

To our knowledge, this is the first study of restenosis after arterial injury in GF mice and includes a novel comparison of vessel histology and morphometry pre- and post-injury. Our methodology of transferring GF mice in/out of sterile isolators in order to perform microsurgery in a biosafety cabinet preserved sterility for up to 28 days. Other investigators recently performed carotid ligation to study thrombosis[68] and middle cerebral artery occlusion to induce stroke[34] in GF mice, which required survival for up to 5 days, respectively. We also identified potential mechanistic pathways which may impact the pro- and anti-inflammatory immune pathways during arterial remodeling, which add to our understanding of the far-reaching impact of commensal microbes on the peripheral vasculature.

We did not measure local arterial or systemic neutrophil or monocyte/macrophage function or quantify local arterial cytokines or chemokines. However, our comparative immunohistochemistry analysis of immune cell markers and multiplex immunoassays of circulating cytokines and chemokines provides an intriguing starting point for a larger study to address in detail the subsets of immune cells and the kinetics of systemic inflammation that may impact arterial remodeling in the absence of commensal microbes. Although immunohistochemistry is useful for assessing morphology, proliferation, and localization of immune cell accumulation and activation, it requires preparation of fixed tissue, which is susceptible to artifact and permits only semi-quantitative analysis. Hence, we suggest that a broader characterization and quantification of myeloid cells in remodeling arteries is necessary to better define the preliminary observations we have made. Furthermore, while we used cell markers to discriminate between M1 and M2 macrophage polarization states, it has become clear that macrophage polarization is not binary but controlled by multiple genes.[69] Thus, a method (*e*.*g*., flow cytometry) which can simultaneously detect genes in the M1/M2 monocyte and macrophage activation spectrum would be very useful. Finally, we did not explore how lack of microbiota affect the myriad of cellular pathways implicated in neointimal hyperplasia, including serine proteases and matrix metalloproteinases,[70] lysosomal cysteinyl cathepsins,[71, 72] toll-like receptor signaling,[73] and endothelial dysfunction.[74] Given emerging evidence that gut microbiota influence blood pressure regulation and vascular function[32] and interact with pattern recognition receptors in multiple disease models,[75] it will be important that future studies address mechanistic pathways impacted by gut microbiota in the arterial remodeling process.

In summary, we demonstrate that commensal microbiota could represent an environmental exposure that promotes restenosis after arterial interventions. Understanding the inflammatory pathways modulated by microbiota could represent therapeutic targets for the prevention and treatment of restenosis.

## Supporting information

S1 Table Quantitative comparison of morphometric parameters of carotid arteries between CONV-R and GF mice 28 days after carotid ligation. (DOCX)

S2 Table Quantitative comparison of staining indices of post-ligation carotid arteries between CONV-R and GF mice at 5 days. (DOCX)

S3 Table Serum cytokine and chemokine concentrations in CONV-R and GF mice at baseline and after carotid ligation. (DOCX)
