# Supplementary material for: Microbiota control acute arterial inflammation and neointimal hyperplasia development after arterial injury
Source: PLoS One. 2018 Dec 6;13(12):e0208426. doi: 10.1371/journal.pone.0208426 (PMC6283560; doi:10.1371/journal.pone.0208426)
Supplement: S1 Table — (DOCX) [file pone.0208426.s001.docx]

**S1 Table. Quantitative comparison of morphometric parameters of carotid arteries between CONV-R and GF mice 28 days after carotid ligation.**

|  | CONV-R (N=4) | GF (N=8) | P value |
| --- | --- | --- | --- |
| Intima area (mm^2^) | .032±.007 | .007±.004 | **.04** |
| Media area (mm^2^) | .052±.002 | .037±.002 | **.01** |
| Intima+media area (mm^2^) | .083±.010 | .047±.006 | **.01** |
| Intima area/(Intima+media area) | .310±.046 | .111±.040 | **.04** |
| Diameter (mm) | .410±.025 | .379±.018 | .11 |
| % thrombosis | 0 | 0 | NS |

Except where indicated, values are expressed as median±SEM. N=5-10 mice per group. P values ≤.05 are in bold. NS, not significant.
