# Supplementary material for: Microbiota control acute arterial inflammation and neointimal hyperplasia development after arterial injury
Source: PLoS One. 2018 Dec 6;13(12):e0208426. doi: 10.1371/journal.pone.0208426 (PMC6283560; doi:10.1371/journal.pone.0208426)
Supplement: S2 Table — (DOCX) [file pone.0208426.s002.docx]

**S2 Table. Quantitative comparison of staining indices of post-ligation carotid arteries between CONV-R and GF mice at 5 days.**

Intima

|  | CONV-R | GF | P value |
| --- | --- | --- | --- |
| Ki67 | 8.3±2.5 | 21.7±4.0 | **.03** |
| CD45 | 23.5±5.3 | 44.4±7.8 | **.003** |
| CD68 | 7.5±2.9 | 15.7±3.8 | .23 |
| CD206 | .6±.2 | 18.2±4.7 | **.005** |
| Arg1 | 0±0 | .14±.09 | .47 |
| NIMP-R14 | 7.7±3.1 | 20.9±4.0 | **.05** |
| Ly6G | 10.3±2.9 | 19.1±4.3 | **.16** |

Media

|  | CONV-R | GF | P value |
| --- | --- | --- | --- |
| Ki67 | 10.7±2.2 | 4.9±2.2 | **.03** |
| CD45 | 5.4±2.4 | 19.0±4.2 | **.007** |
| CD68 | 1.2±.7 | 6.5±1.6 | **.02** |
| CD206 | .2±.1 | 3.3±1.5 | **.002** |
| Arg1 | .7±.3 | 2.1±.5 | **.004** |
| NIMP-R14 | 1.9±1.2 | 10.5±2.7 | **.03** |
| Ly6G | 21.1±8.1 | 10.5±2.9 | .91 |

Adventitia

|  | CONV-R | GF | P value |
| --- | --- | --- | --- |
| Ki67 | 18.4±2.1 | 8.9±1.2 | **.01** |
| CD45 | 27.3±1.3 | 40.1±3.6 | **<.001** |
| CD68 | 23.4±1.1 | 24.9±1.3 | .46 |
| CD206 | 22.9±1.1 | 28.4±1.6 | **.02** |
| Arg1 | 9.4±2.1 | 12.8±2.1 | **.05** |
| NIMP-R14 | 3.0±.6 | 6.1±1.0 | **.04** |
| Ly6G | 10.1±1.6 | 10.4±1.6 | .83 |

Values are expressed as mean±SEM. N=6-8 mice per group. P values ≤.05 are in bold.
