# Supplementary material for: Microbiota control acute arterial inflammation and neointimal hyperplasia development after arterial injury
Source: PLoS One. 2018 Dec 6;13(12):e0208426. doi: 10.1371/journal.pone.0208426 (PMC6283560; doi:10.1371/journal.pone.0208426)
Supplement: S3 Table — (DOCX) [file pone.0208426.s003.docx]

**S3 Table. Serum cytokine and chemokine concentrations in CONV-R and GF mice at baseline and after carotid ligation**

|  | Baseline | | | 5 days | | | 28 days | | |
| --- | --- | --- | --- | --- | --- | --- | --- | --- | --- |
| Cytokine or chemokine  (pg/ml) | **CONV-R** (n=5) | **GF**  (n=4) | **P value** | **CONV-R** (n=13) | **GF**  (n=8) | **P value** | **CONV-R** (n=4) | **GF**  (n=5) | **P value** |
| IL-1β | .25±.19 | 0±0 | .44 | 1.87±.52 | 2.60±1.20 | .60 | .97±.62 | .62±.32 | .79 |
| IL-2 | 1.91±1.91 | 0±0 | >.99 | 2.63±1.57 | 0±0 | .24 | .96±.75 | 0±0 | .17 |
| IL-4 | **4.59±.89** | **.82±.82** | **.03** | 7.43±.36 | 8.99±.81 | .09 | 7.44±1.07 | 6.80±.72 | .90 |
| IL-5 | 13.59±6.64 | 2.41±1.66 | .17 | 21.52±4.07 | 20.91±2.57 | .47 | 14.63±1.76 | 12.64±2.08 | .51 |
| IL-6 | 0±0 | 0±0 | >.99 | 34.04±9.45 | 9.31±3.62 | .10 | 1.86±.72 | 1.19±1.94 | .36 |
| IL-9 | 17.39±17.39 | 27.01±11.89 | .44 | 12.43±5.12 | 29.38±11.26 | 027 | 11.67±8.78 | 17.36±17.36 | >.99 |
| IL-10 | 0±0 | 0±0 | >.99 | 23.73±8.53 | 9.26±3.07 | .80 | 3.59±1.20 | 2.91±1.85 | .76 |
| IL-12p70 | .85±.85 | 0±0 | >.99 | **0.38±0.23** | **1.75±.57** | **.005** | 1.34±0.22 | 1.38±.25 | .90 |
| IL-13 | 0±0 | 0±0 | >.99 | 0±0 | 0±0 | >.99 | 0±0 | 0±0 | >.99 |
| IL-17a | 0±0 | 0±0 | >.99 | 0±0 | 0±0 | >.99 | 0±0 | 0±0 | >.99 |
| IL-18 | **70.36±11.71** | **0±0** | **.04** | 217.50±22.16 | 287.60±29.58 | .34 | 152.60±21.84 | 210.2±90.41 | .68 |
| IL-22 | 6.50±3.35 | 4.95±4.42 | .87 | 61.90±19.55 | 26.32±10.86 | .37 | 26.41±6.34 | 8.04±3.52 | .063 |
| IL-23 | 7.37±7.37 | 15.14±5.80 | .30 | 61.27±22.34 | 38.64±14.57 | .82 | 17.94±2.43 | 10.18±4.91 | .17 |
| IL-27 | 18.99±9.10 | 5.86±2.04 | .41 | **38.66±5.42** | **13.24±2.67** | **.001** | 33.49±8.01 | 13.53±7.00 | .11 |
| IFN-ɣ | .54±.40 | .47±.44 | .70 | 2.54±.65 | 3.15±.99 | .79 | 1.73±.52 | 3.57±1.04 | .19 |
| TNF-α | 0±0 | 0±0 | >.99 | 2.53±.94 | .96±.90 | .30 | 0±0 | 1.04±1.04 | >.99 |
| Eotaxin | 1520±357.90 | 1168±51.23 | .85 | 2862±271 | 2320±315.5 | .24 | 2316±72.94 | 1993±184.4 | .41 |
| GM-CSF | 0±0 | 0±0 | >.99 | 0±0 | 0±0 | >.99 | 0±0 | 0±0 | >.99 |
| Gro-alpha/KC | 52.46±14.1 | 27.33±4.87 | .11 | 124.4±35.83 | 112±24.86 | .75 | 52.4±9.31 | 65.66±10.35 | .41 |
| IP-10 | 31.47±6.92 | 45.51±17.88 | .89 | **125.8±13.42** | **67.02 ± 10.27** | **.004** | 77.83±14.82 | 68.95±11.72 | .73 |
| MCP-1 | 49.17±10.11 | 47.03±3.09 | .41 | **117.9±19.55** | **57.53±8.61** | **.05** | 60.11±4.36 | 50.56±6.25 | .29 |
| MCP-3 | 146.7±20.5 | 125.6±21.98 | .730 | 408.3±65 | 311.4±25.23 | .64 | 144.2±5.88 | 146.9±29.84 | >.99 |
| MIP-1α | 0±0 | 0±0 | >.99 | .27±.27 | .21±.11 | .11 | .05±.05 | 2.39±2.38 | .84 |
| MIP-1β | .43±.43 | .72±.72 | .72 | 2.94±.47 | 3.23±.64 | .75 | 2.69±.55 | 2.48±.28 | .52 |
| MIP-2 | 36.59±5.84 | 35.49±6.08 | >.99 | **43.41±2.74** | **56.99±3.52** | **.006** | 60.21±1.26 | 41.63±3.32 | .17 |
| RANTES | 13.94±11.07 | 20.73±8.02 | .44 | 34.23±5.51 | 52.65±12.20 | .27 | 54.47±10.75 | 55.62±17.90 | .56 |

Comparisons with P<.05 are shown in bold.
